# Taura Syndrome Virus and Mammalian Cell Lines

**DOI:** 10.3201/eid1012.040537

**Published:** 2004-12

**Authors:** Peng Luo, Chao-Qun Hu, Chun-Hua Ren, Zhao-Feng Sun

**Affiliations:** * The Chinese Academy of Sciences, Guangzhou, People's Republic of China

**Keywords:** letter, shrimp virus, viral infection, mammalian cell lines

**To the Editor:** Audelo-del-Valle et al. concluded that human and monkey cell lines (rhabdomyosarcoma [RD], human larynx carcinoma [Hep-2C], and Buffalo green monkey kidney [BGM]) could be infected by a penaeid shrimp virus, Taura syndrome virus (TSV) (*1*). They also concluded that *Penaeus* spp. could likely be a reservoir of a virus that might become pathogenic to humans and other mammals (*1*).

Though researchers have tried to develop continuous marine crustacean cell lines for >30 years, their efforts have not been successful. The lack of continuous marine crustacean cell lines has become an obstacle to conducting research on viral disease in shrimp (*2**,**3*). During the last 20 years, many researchers searched for substitute cell lines on which to study shrimp viruses (*4**,**5*). Audelo-del-Valle et al. likely chose RD, Hep-2C, and BGM cell lines because TSV was "recently reported to be genomically related to the cricket paralysis virus of the *Cripavirus* genus, family *Dicistrovirdae* of the 'picornavirus superfamily'" (*1*), and these cell lines were susceptible to some picornaviruses. If their findings are correct, they may have found substitute cell lines for isolating and studying TSV. To confirm their findings, we selected two mammalian cell lines, Hep-2 and Vero, which are highly sensitive to some picornaviruses (*6**,**7*), and tested them to determine their susceptibility to TSV.

The TSV extract was prepared from frozen cephalothoraxes of shrimp, *Litopenaeus vananmei*, that were infected with TSV (confirmed by standard reverse transcriptase–polymerase chain reaction [RT-PCR]) (*8*). To verify the TSV extract's validity, 50 μL of diluted TSV extract (approximately 0.8% volume of shrimp body weight) was injected into each of eight healthy shrimp, *L. vannamei*. Another eight healthy shrimp (control group) were injected with a diluted extract prepared from frozen cephalothoraxes of healthy shrimp. All of the TSV-injected shrimp died within 6 days and were TSV-positive; control shrimp did not die and were TSV-negative, which showed that our TSV extract was active and viable. The TSV extract was transferred into cell culture flasks according to a method previously reported (*9*). The cell monolayers were exposed to 100 μL of diluted and filtered TSV extracts for 1 hour; the extracts were then removed from the flasks, 2 mL of maintenance medium was added to each flask, and the flasks were incubated in three separate rooms at 37°C, 35°C, and 33°C, respectively. If a cytopathic effect (CPE) was not evident within 7 days, cell monolayers were washed with Hank's balanced salt solution (HBSS) six times to eliminate viral particles from the primary extract or from infected cells. Then cells were lysed in 2 mL HBSS, the lysate was clarified, and a portion of it was used for the first passage. This procedure was repeated three times. The control cell lines were injected with diluted extract from healthy shrimp, and passage was conducted as described earlier. RNA samples were extracted and purified from 150-μL lysates of primary cells and four passage cells and used as templates for RT-PCR analysis to determine the presence of TSV.

No CPE was observed in either the Hep-2 or Vero cell line that had been injected with TSV after 7 days of culture at any of the three temperatures tested, and CPE was not found after the fourth passage. The RT-PCR analysis resulted in weak amplification (positive) from the first lysate, but no amplification was found in lysates of four passage cells. Had TSV replicated (productive infection) in either of the two cell lines, RT-PCR would have shown a strong amplification from each lysate. Such a weak amplification may have been the result of residual extracellular viruses that remained in the cell culture flask after washing. However, after first passage and repeated washing with HBSS, any remnants of the original medium were not likely to have been present. Therefore, our result showed that TSV was incapable of infecting Hep-2 and Vero cell lines.

Generally, aquatic viruses replicate in cells of aquatic animals at 20°C–35°C, their natural environmental temperature. We incubated cultures as noted earlier, as we did not know which temperature was most conducive for viral replication; all attempts were unsuccessful. Hep-2 and Hep-2C derive from the same tissue (human Caucasian larynx carcinoma), while Vero and BGM derive from another organ (Africa green monkey kidney). Thus, Hep-2 and Vero cell lines that we used are likely susceptible to TSV if the virus can infect Hep-2C and BGM as reported by Audelo-del-Valle et al.

The difference between our methods and those used by Audelo-del-Valle et al. may explain the discrepant result. If CPE occurred "usually from 19–23 hours" and "cells were then harvested and lysed" for next injection, TSV most likely persisted in the lysate after the third passage because the cell monolayers had not been washed as they were in our method. According to the time the CPE was observed and the methods of Audelo-del-Valle et al., we assumed that, in their study, cells might be passaged at least three times within 1 week, whereas TSV might remain viable and infective for 1 week. Additionally, the Office International des Epizooties recommended an injection volume of 1% of shrimp body weight (*8*); Audelo-del-Valle et al. used 10%. With such a large dose, shrimp could be infected easily with TSV from the initial medium and die suddenly. Moreover, the evidence of successful infection from photos of CPE only is not sufficient; Audelo-del-Valle et al. should offer more convincing evidence from images of viral particles in cells by electron microscope or in situ hybridization. Therefore, we think the CPE that Audelo-del-Valle et al. reported was not caused by TSV but by a virus contaminant or some harmful component from shrimp extract.

The structure of the TSV genome is similar to that of small insect–infecting RNA viruses (*10*), which belong to a renamed virus genus, *Cripavirus* (*11*). No published reports have shown that other viruses in this genus are able to infect mammalian cells or cell lines. Moreover, TSV is prevalent in shrimp farming areas in the world, and *L. vannamei* (principal host for TSV) are eaten by people worldwide (*8*). In China, some persons eat fresh shrimp without disinfecting them; however, no evidence shows that TSV can infect humans. The results of our study show that TSV cannot infect mammalian cell lines or cells.
